# Inhibitor Trapping in Kinases

**DOI:** 10.3390/ijms25063249

**Published:** 2024-03-13

**Authors:** Danislav S. Spassov, Mariyana Atanasova, Irini Doytchinova

**Affiliations:** Drug Design and Bioinformatics Lab, Department of Chemistry, Faculty of Pharmacy, Medical University of Sofia, 1000 Sofia, Bulgaria; matanasova@pharmfac.mu-sofia.bg (M.A.); idoytchinova@pharmfac.mu-sofia.bg (I.D.)

**Keywords:** kinases, binding affinity, Src, Abl, imatinib, drug design, binding affinity prediction, protein conformation, drug potency

## Abstract

Recently, we identified a novel mechanism of enzyme inhibition in N-myristoyltransferases (NMTs), which we have named ‘inhibitor trapping’. Inhibitor trapping occurs when the protein captures the small molecule within its structural confines, thereby preventing its free dissociation and resulting in a dramatic increase in inhibitor affinity and potency. Here, we demonstrate that inhibitor trapping also occurs in the kinases. Remarkably, the drug imatinib, which has revolutionized targeted cancer therapy, is entrapped in the structure of the Abl kinase. This effect is also observed in p38α kinase, where inhibitor trapping was found to depend on a ‘magic’ methyl group, which stabilizes the protein conformation and increases the affinity of the compound dramatically. Altogether, these results suggest that inhibitor trapping is not exclusive to N-myristoyltransferases, as it also occurs in the kinase family. Inhibitor trapping could enhance the binding affinity of an inhibitor by thousands of times and is as a key mechanism that plays a critical role in determining drug affinity and potency.

## 1. Introduction

We recently described a mechanism in N-myristoyltransferases (NMTs)—inhibitor trapping [1]. Inhibitor trapping relies on NMTs’ inherent ability to transition between open and closed conformations, a crucial process for accommodating substrate molecules, catalyzing the enzymatic reaction, and releasing reaction products [1,2]. Our findings suggest that the potency of NMT inhibitors depends on their ability to stabilize the enzyme conformation in the closed state. In this state, the small molecules are trapped and locked inside the enzyme’s structural confines, creating a substantial barrier to their dissociation. A central role played by inhibitor trapping in NMTs involves its Ab-loop, which is positioned just above the drug binding site and can adopt open and closed conformation, regulating the accessibility of the catalytic center. Molecular dynamics simulations have indicated that the inhibitor gains access to the binding site when the Ab-loop is open, and its closure captures the drug inside the protein structure. Interactions between the inhibitor and the amino acid residues at the base of the Ab-loop prevent its reopening, effectually locking and trapping the small molecule inside the enzyme catalytic center [1].

A key hallmark of inhibitor trapping is the high affinity of the interaction, resulting primarily from very low dissociation rates [1]. The persistence of the inhibitor trapping state is contingent on the reduction in protein dynamics, as the dissociation of the inhibitors necessitates conformational changes within the protein structure to release the small molecule [1]. An outcome of the inhibitor entrapment is the exceptional influence of the bonds that capture the small molecule. These bonds contribute to the enhancement of the affinity or activity of the compounds hundreds or even thousands of times, significantly exceeding their anticipated contributions to binding energy [1,3]. As a result, the conformational transitions observed in protein–ligand complexes, as determined through MD simulations, were found to reflect the efficiency of inhibitor entrapment and correlate with the binding affinity and activity of the compounds [1].

A fundamental question that remains unanswered in the studies of NMTs is whether the mechanism of inhibitor entrapment is specific to these enzymes, making it an exception rather than a rule. To address this, we investigated the possibility that other proteins might also be inhibited through inhibitor trapping. Our focus turned to protein kinases. The protein kinase family consists of over 500 members and has been extensively studied and recognized as one of the most promising pharmaceutical targets [4,5,6,7].

**Figure 1:**
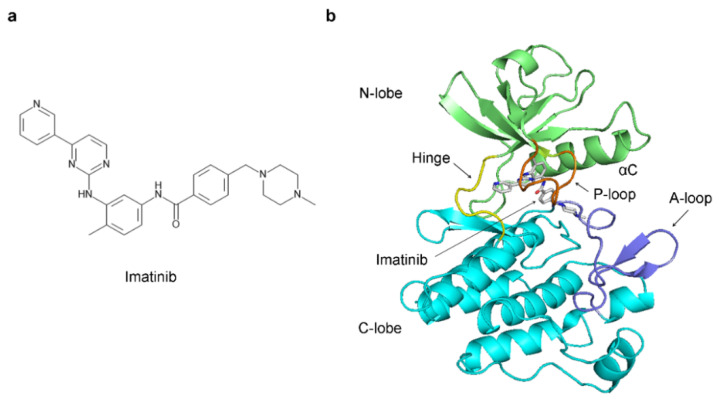
The structure of Abl in a complex with imatinib: (**a**) 2D structure of imatinib; (**b**) crystal structure of Abl in a complex with imatinib (PDB 1IEP). The Abl N-lobe is shown in green, the C-lobe in cyan, and the hinge region in yellow. The P-loop and A-loop are indicated in orange and blue. The carbon atoms of imatinib are shown in grey.

Throughout history, research into protein kinases has played a pivotal role in shaping our comprehension of oncological processes. Notably, the identification of the first oncogene, the protein kinase Src, within the Rous sarcoma virus (RSV) and the subsequent discovery of its cellular counterpart, c-Src, marked the inception of the oncogene theory of cancer development [8,9]. This transformative journey continued with the recognition of a homologue of Src in the Abelson murine leukemia virus, known as Abl [10]. Its cellular equivalent, c-Abl, was later found to be involved in Philadelphia chromosome translocation, leading to the formation of the fusion Bcr-Abl protein responsible for the development of Chronic Myeloid Leukemia (CML) [11]. The advent of imatinib (Figure 1a), a selective Abl kinase inhibitor also marketed as Gleevec, resulted in lasting cancer remission in CML patients, demonstrating, for the first time, a highly successful targeted therapy for cancer [12,13]. The protein kinases use ATP to phosphorylate the hydroxyl groups of specific serine and threonine residues (serine/ threonine kinases) or tyrosine residues (protein tyrosine kinases) [14]. The phosphorylation events function as on/off switches for the phosphorylated protein or serve as docking sites to assemble macromolecular complexes necessary for the transduction of the cell signaling events [6].

The kinase domain within the kinases comprises two essential components: the smaller N-lobe and the larger C-lobe, interconnected by a flexible linker known as the hinge region (Figure 1b) [15].

The hinge’s conformational flexibility permits the N-lobe and C-lobe to move in relation to one another, thereby governing the dynamic opening and closing of the kinase domain. The catalytic center of the kinases is located at the cleft interface between the two lobes and compromises a region known as the ATP binding pocket, where the cofactor ATP binds [15]. On top of the ATP binding pocket is the phosphate-binding loop, more frequently abbreviated as the P-loop (Figure 1b) [16]. The P-loop is essential for ATP binding and subsequent transfer of phosphate groups to target proteins during phosphorylation events. The HRD (His-Arg-Asp) motif is located in the catalytic loop and also critically involved in coordinating and stabilizing ATP binding. The activation loop, also known as the A-loop, regulates the entry of the substrate peptide into the catalytic center and the activity of the kinases (Figure 1b) [17]. At the beginning of the A-loop, there is the conserved DFG motif (Asp-Phe-Gly) [18]. In the active conformation of the kinase domain, the aspartic acid from this motif coordinates a magnesium ion, which is essential for ATP binding and phosphate transfer during substrate phosphorylation [18]. Another critical residue for the catalysis is the glutamic acid, located in the αC-helix (Figure 1b), which forms a salt bridge with a conserved lysine residue in the active site [19]. Mutating these residues completely abrogates the kinase activity [4]. 

The protein kinases are known to adopt different conformations [20]. The active, catalytically competent conformation is only one for all kinases because only one conformation allows the correct positioning of the amino acid residues in the active site to allow the phosphorylation reaction [20]. In this active conformation, the aspartic acid from the DFG loop faces toward the catalytic center (DFG-in conformation) and interacts with the magnesium ion, which is essential for catalysis [20]. In the active conformation, the glutamic acid from the αC- helix also faces toward the catalytic center and forms a salt bridge with the conserved lysine residue [20]. In contrast, the kinases can adopt different inactive conformations [20]. In the complexes of Src and Abl with imatinib, the aspartic acid from the DFG motif is flipped, and its side chain is facing outward the catalytic center (DFG-out conformation), while the αC-helix remains in the active position [21]. Conversely, in some other crystal structures, it has been observed that the αC-helix, together with its conserved glutamic acid, moves away from the catalytic center. This αC-helix-out conformation is also known as CDK/Src inactive conformation because it was first observed in cyclin-dependent kinases and c-Src [20]. 

The success of imatinib for the treatment of CML has prompted significant interest in the mechanism of its activity. It was recognized that one of the reasons for its success is its high selectivity for Abl kinases over other kinases, including its closest homologue, c-Src [22]. Although imatinib displays a 3000-fold higher affinity for Abl than for Src, the crystal structure of the Abl and Src complexes with imatinib has unveiled a remarkable similarity; the binding sites for imatinib on Src and Abl are nearly identical, raising the question of how imatinib achieves its selectivity [22]. This puzzling problem has been the subject of extensive research for over two decades, giving rise to a succession of hypotheses and theories that continue to evolve with the emergence of new evidence [23,24]. 

Here, we examined whether imatinib selectivity for Abl over Src can be explained through the mechanism of inhibitor trapping, considering that a nearly identical situation has been observed in N-myristoyltransferases, where a selective inhibitor was developed for protozoan NMTs over the human NMTs [1,25]. This compound has a 215-fold higher affinity for NMTs from *Leishmania major* than the human NMTs, however due to the evolutionary conservation of the catalytic domain, the inhibitor shows an identical binding mode and forms exactly the same interactions within the binding sites of the two proteins [25]. In this case, the difference in affinity could be explained by the altered protein dynamics of the two proteins that determine how efficiently the ligand is trapped into the protein structure.

A defining characteristic of inhibitor trapping is that the bonds involved in the ligands’ entrapment have a very high contribution to overall binding affinity [1,3]. A literature search identified many such examples, including the intriguing phenomenon known as the ‘magic’ methyl effect, where the addition of a single methyl group to a compound enhances its affinity hundreds or even thousands of times, instead of the expected 3.5-fold increase, due to the hydrophobic interaction of the methyl group with the protein [26,27,28]. This ‘magic’ methyl effect has been observed across various proteins, including kinases. In kinases, it has been reported that the introduction of a methyl group to an inhibitor of p38α kinase substantially amplifies its potency by over 208-fold [27], prompting us to suspect another instance of inhibitor trapping.

In this study, we conducted analyses of Abl and Src imatinib complexes, as well as the ‘magic’ methyl effect in the p38α. The results demonstrate that these intriguing phenomena can be explained through the concept of inhibitor trapping, and they appear to share a common underlying mechanism involving the P-loop of the kinase domain.

## 2. Results

### 2.1. Inhibitor Trapping in p38α

**Figure 2:**
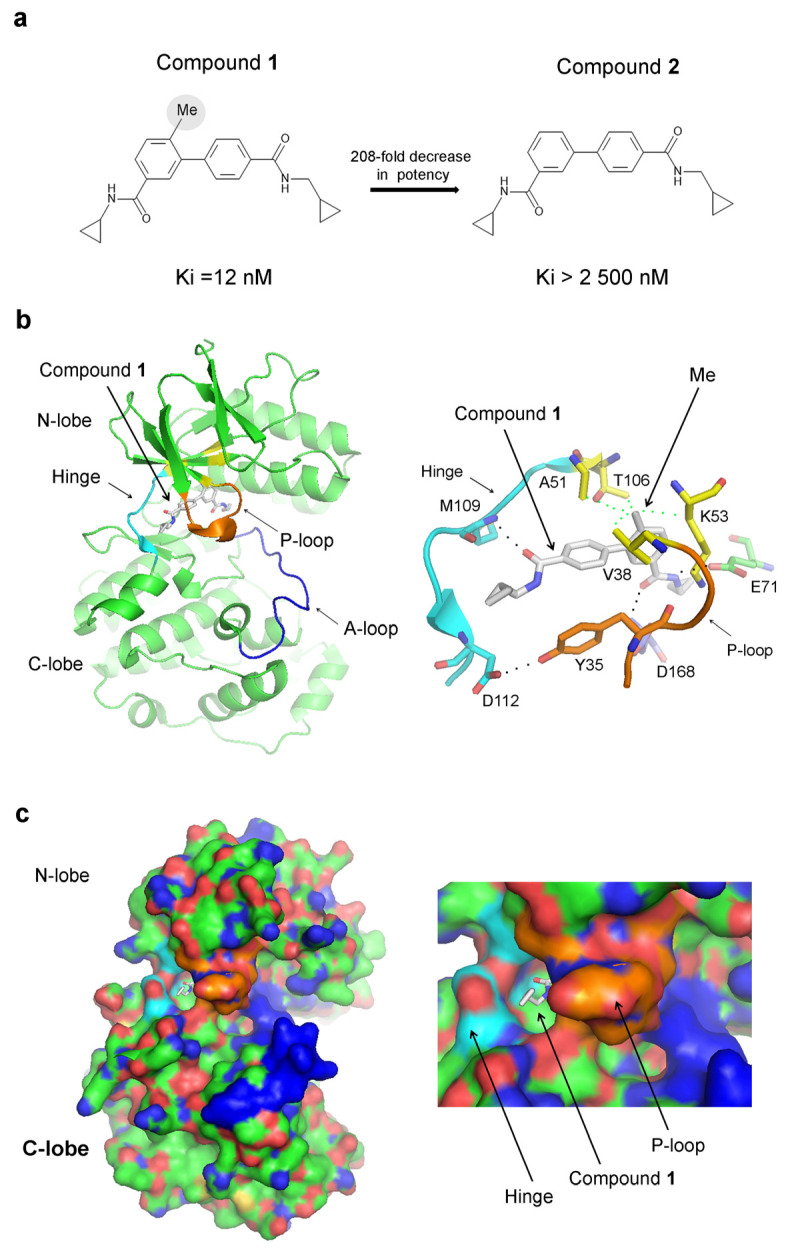
Inhibitor trapping in p38α: (**a**) The 2D structure of compounds **1** and **2**. The removal of the indicated methyl group (grey circle) leads to a 208-fold reduction in potency. (**b**) The crystal structure of p38α in a complex with compound **1** (PDB 3D7Z). The image on the left shows the whole catalytic domain, and on the right, the binding site of compound **1** is shown. The P-loop, which adopts a closed conformation, is shown in orange. The A-loop is in blue, the hinge region is in cyan, and the hydrophobic pocket, where the ‘magic’ methyl group interacts, is in yellow. (**c**) Surface representation of the crystal structures depicted in “(**b**)”with the corresponding coloring scheme, showing that compound **1** is captured inside the p38α protein structure.

The addition of an extra methyl group boosts the affinity of an inhibitor of p38α—compound **1**—by over 208-fold compared to compound **2**, where the methyl group is absent (Ki = 12 nM for compound **1** and Ki > 2500 nM for compound **2**), (Figure 2a) [27]. The methyl group has a surprisingly robust effect on affinity, given that its theoretical contribution is estimated to be just 3.5-fold [27]. The crystal structure of p38α in a complex with compound **1** (PDB 3D7Z) reveals that the P-loop adopts a closed conformation (Figure 2b). In this conformation, a tyrosine residue Tyr35 from the P-loop interacts with Asp112 in the hinge region, effectively enclosing the small molecule (Figure 2b). Other interactions include hydrogen bonds with Met109 in the hinge region, Asp168 from the DFG motif, and Glu71 from the αC-helix. The methyl group of compound **1** is positioned within a hydrophobic pocket, which is shaped by Val38, Ala51, Lys53 (the aliphatic carbon atoms of the lysine side chain comprises the pocket, while the positively charged amino group remains external), and Thr106 (Figure 2b). Lys53 represents the conserved amino acid that forms a salt bridge with Glu71 from the αC-helix in the active kinase conformation required for kinase activity, while Thr106 is the gatekeeper residue. The closed conformation of the P-loop suggests that compound **1** could be trapped inside the protein structure. Indeed, a surface representation of the crystal structure of p38α in a complex with compound **1** reveals that the inhibitor is enclosed within the protein structure, with only a minor portion of the molecule being exposed to the solvent (Figure 2c). The hydrogen bond between Tyr35 and the hinge region stabilizes the closed P-loop conformation, sealing the inhibitor from the environment (Figure 2b,c).

**Figure 3:**
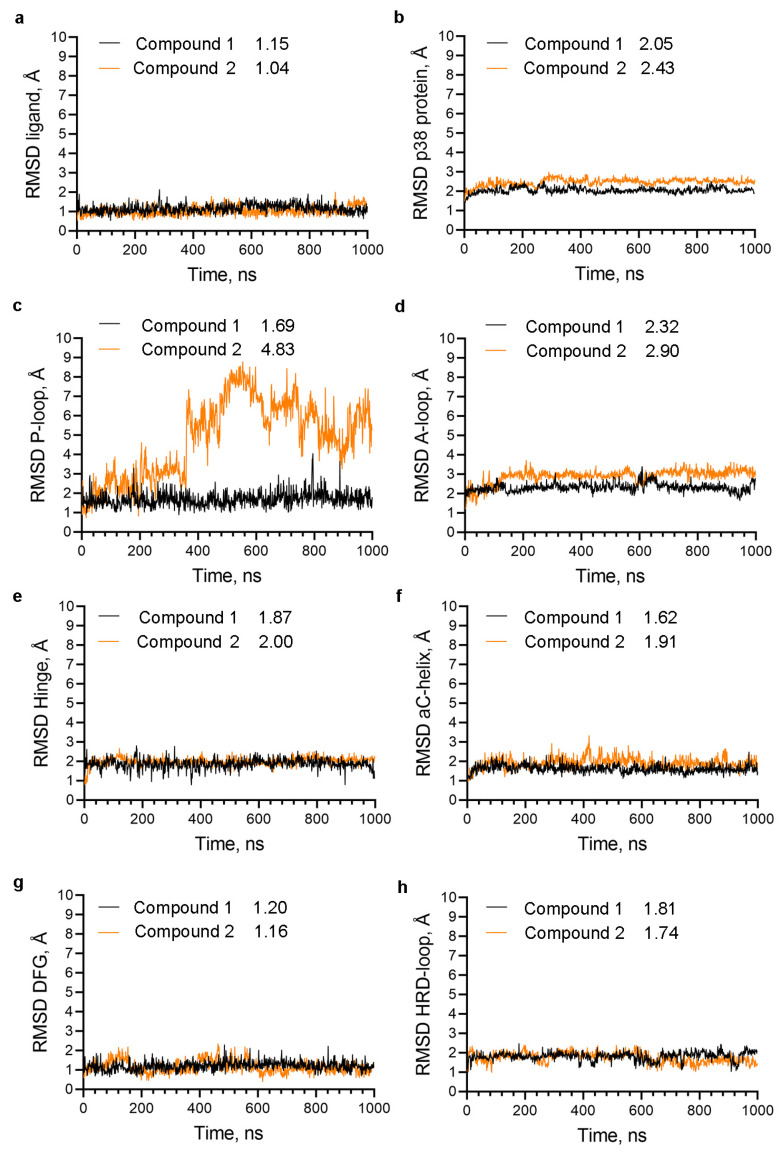
The role of the ‘magic’ methyl group in stabilizing the P-loop conformation: (**a**–**h**) RMSD of the heavy atoms in p38α complexes with compound **1** and compound **2** (without methyl) of the indicated structural elements. The numbers next to the legend indicate the average RMSD in Angstroms (Å).

Given that the release of the trapped inhibitors requires a conformational change in the protein structure, we conducted MD simulations of p38α complexes with either compound **1** (PDB 3D7Z), containing the methyl group, or compound **2**, from which the methyl group has been removed (Figure 3). The RMSD values of the ligands—compound **1** and compound **2**—remain low and near identical (Figure 3a); hence, they do not predict the compound’s affinity. The absence of the methyl group leads to an increase in the protein dynamics (Figure 3b). The most striking difference occurs in the RMSD of the P-loop, indicating significant conformational movement in this region (Figure 3c).

The absence of the methyl group also increases the dynamics of the A-loop, although not as substantially as the P-loop (Figure 3d), and hence, it does not indicate a sizeable conformational shift in this region. The conformation of the p38α kinase domain in a complex with compound **1** is αC-helix–in, DFG-in, and this conformation is pertained during the MD simulations, as evidenced by the low RMSD values for these regions (Figure 3f,g). No substantial conformational changes are observed in the hinge region and the catalytic loop containing the HRD motif (Figure 3e,h). 

Since other regions in the p38α protein may show altered dynamics, we performed RMSD analysis per amino residue (Figure S1). This analysis has confirmed the large conformational change in the P-loop (Figure S1). In addition, it identified L16—the loop between αI and αL-helices—as a region of increased dynamics (Figure S1). This region connects to αL-helix, which, although C-terminal in the primary sequence, associates with the N-lobe of the kinase domain. However, the role of this region in mediating affinity, if any, is more challenging to understand, as it is not part of the binding site. 

**Figure 4:**
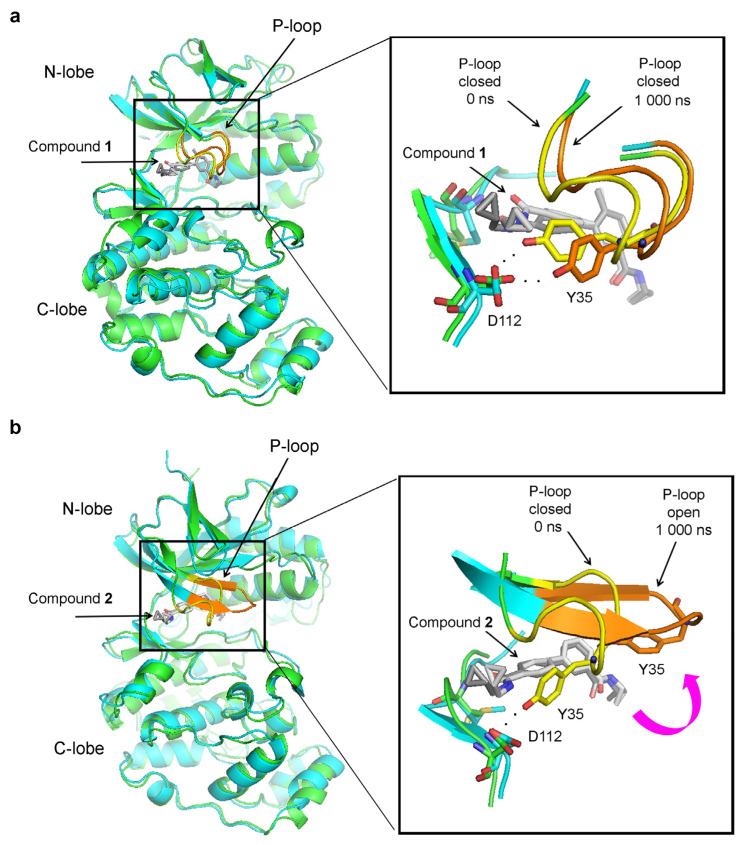
Conformational changes in the P-loop observed during MD simulations of p38α complexes. Structural superimpositions of the structures at the beginning (0 ns) and the end (1000 ns) of the MD simulations are shown. The images on the left show the whole p38α kinase domain, and on the right the images depict zoomed views of the boxed areas. In time 0, the protein is shown as a green cartoon, with the P-loop in yellow and the inhibitor in dark grey. In time 1000 ns, the protein is in cyan, the P-loop is in orange, and the ligand is in light grey: (**a**) the complex between p38α and compound **1**- the P-loop preserved its closed conformation during MD simulations. (**b**) the complex between p38α and compound **2** (without a methyl group). The P-loop transitions from the closed to open conformation, leading to disruption of the interaction between Y35 from the P-loop and D112 in the hinge region.

Considering the dramatic change in RMSD values in the P-loop, we investigated how its conformation changed during the MD simulations. In the p38α complex with compound **1**, the P-loop is closed at the beginning of the MD simulations and remains as such at the end of the simulations (Figure 4a). However, in the absence of the methyl group, the P-loop transitions to an open conformation (Figure 4b). In this open conformation, Y35 no longer interacts with the hinge region and moves away, exposing the small molecule to the solvent (Figure 4b). The P-loop opens at around 360 ns in the simulation and remains open to its end (Figure 3c).

### 2.2. Inhibitor Trapping in Abl Kinase

**Figure 5:**
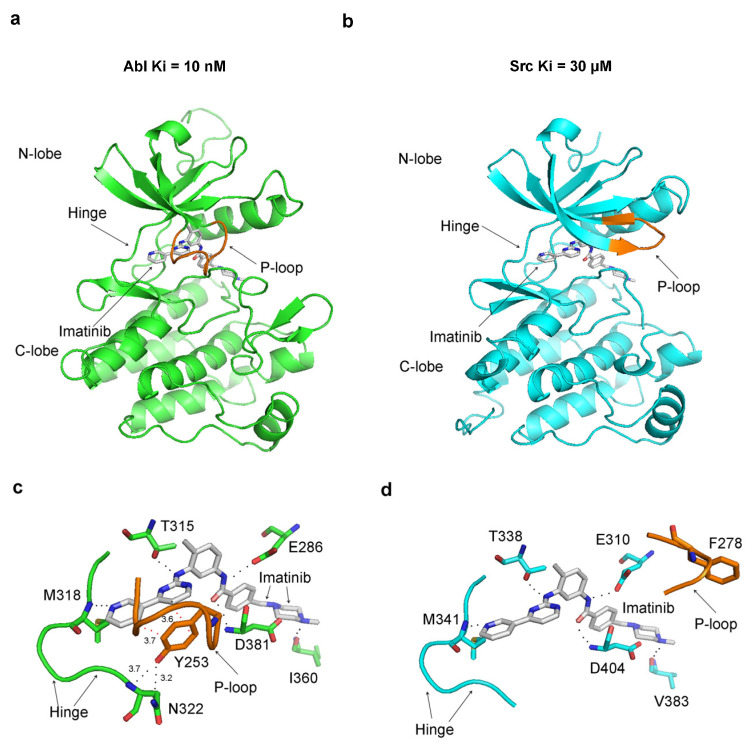
The P-loop adopts different conformations in Src and Abl complexes with imatinib. Images on top depict the whole kinase domain, and at the bottom, they depict the imatinib binding site. The dissociation constants Ki for imatinib with Abl and Src are shown on top: (**a**,**c**) crystal structure of Abl in a complex with imatinib (PDB 1IEP); (**b**,**d**) crystal structure of Src in a complex with imatinib (PDB 2OIQ). All polar interactions (black dots) are conserved in the two complexes. I360 in Abl corresponds to V383 in c-Src, D381 to D404, E286 to E310, T315 to T338, and M318 to M341. In Abl, imatinib forms stacking interactions with Y253 (red dots), which do not occur with the corresponding F278 in c-Src due to the different conformation of the P-loop. In Abl, Y253 also forms a hydrogen bond with N322 from the hinge region. The distances shown are in Angstroms (Å).

Imatinib has a 3000-fold higher affinity for the Abl protein over Src (Ki = 10 nM for Abl, and Ki = 30 µM) [23,24], a characteristic that remains enigmatic given the strikingly similar binding patterns observed in the crystallographic structures of their complexes. One key difference in the crystal structures of Src and Abl bound to imatinib is the conformation of the P-loop. In the Abl–imatinib complex, the P-loop is closed (referred to in the literature as kinked), contrasting with the open (stretched) conformation of the P-loop in Src (Figure 5a,b). More detailed analysis reveals that in Abl, Tyr253 from the P-loop (analogous to Tyr35 in p38α) stacks with the inhibitor’s aromatic rings and forms a hydrogen bond with Asn322 from the hinge region (Figure 5c). In Src, the P-loop is in the open conformation, and Phe278 (the corresponding residue to Tyr253) moves far away from the binding site (Figure 5d). 

**Figure 6:**
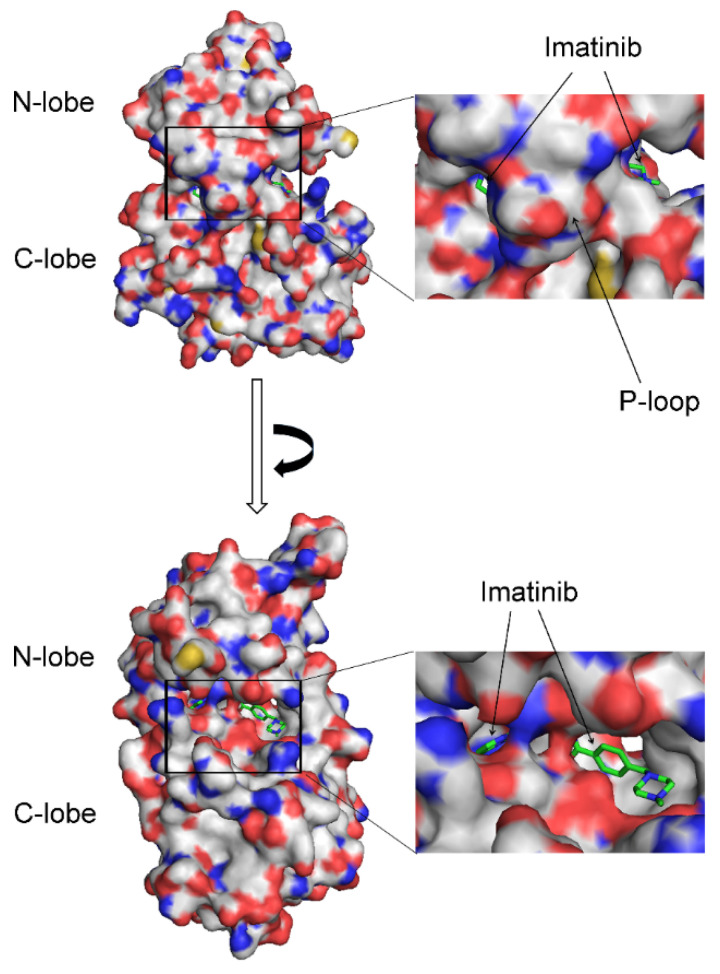
Imatinib trapping inside the structure of Abl. The surface representation of Abl’s crystal structure in a complex with imatinib (PDB 1IEP) is depicted in two different orientations. The carbon atoms of imatinib are shown in green, and the Abl protein is shown in grey. Blue and red correspond to electropositive and electronegative atoms.

In the crystal structure of the Abl–imatinib complex, the P-loop folds on top of the inhibitor, and the hydrogen bonding with the hinge region seals the inhibitor inside the structure of the protein. A surface representation of the Abl complex with imatinib reveals that the inhibitor is captured and bound inside the enzyme’s structure, with the closed P-loop sealing it from the solvent environment (Figure 6). 

In Src, the P-loop’s open conformation does not provide as tight an enclosure around the inhibitor. These findings imply that in Abl, the closed conformation of the P-loop could be involved in capturing and trapping imatinib within its protein structure.

**Figure 7:**
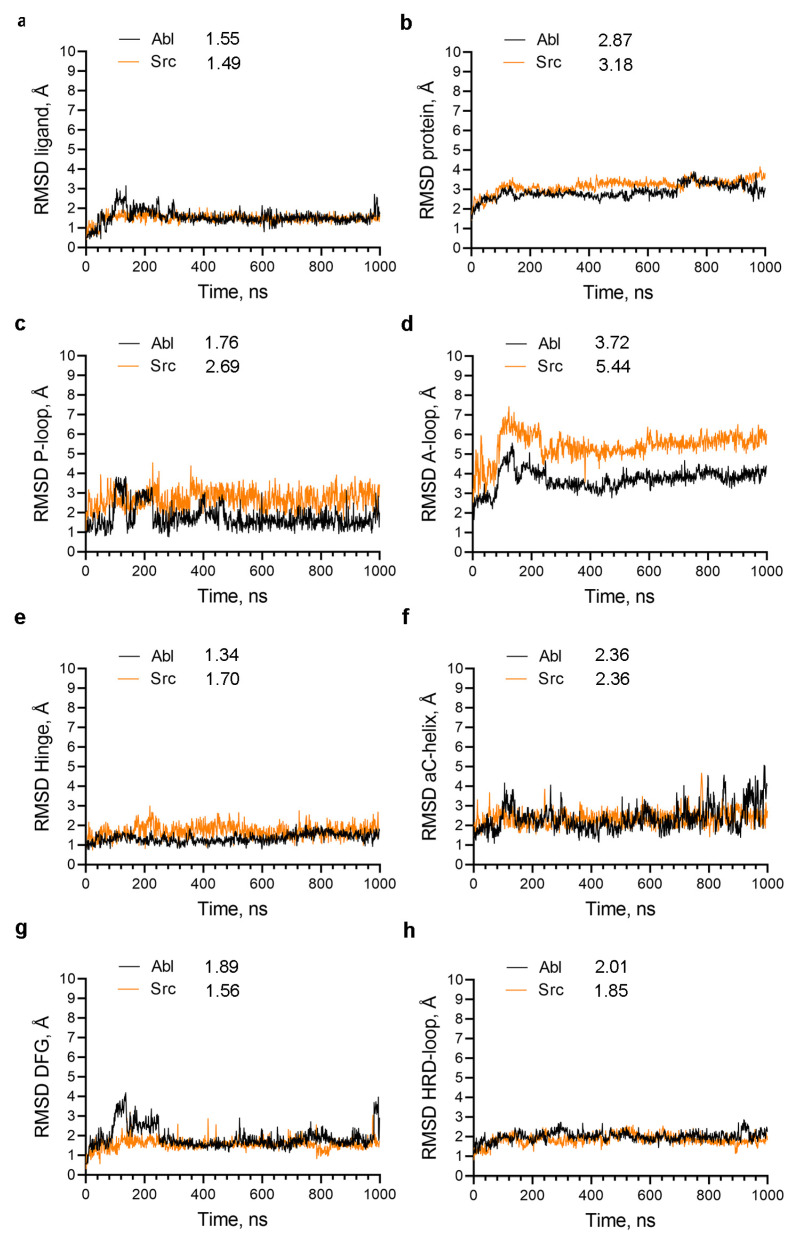
Conformational dynamics of the Abl and Src complexes with imatinib: (**a**–**h**) RMSD of the heavy atoms of the indicated structural elements in Abl and Src complexes with imatinib during MD simulations. The numbers on the legend’s right indicate the average RMSD in Angstroms (Å).

Next, we conducted molecular dynamics (MD) simulations of the imatinib complex with Abl (PDB 1IEP) or Src (PDB 2OIQ) (Figure 7). In these complexes, the RMSD of imatinib is low and exhibits a slight increase in the Abl complex compared to Src (Figure 7a); hence, it is not correlated with the drug’s affinity. This aligns with our prior findings in N-myristoyltransferases, showing that ligands’ RMSD values during MD simulations do not predict their activity. 

The dynamics of the Src protein is increased compared to Abl in their complexes with imatinib (Figure 7b), mainly due to increased dynamics of the P-loop and the A-loop (Figure 7c,d). Examination of the P-loop dynamics indicates that it does not undergo conformational transitions during MD simulations, e.g., in Abl, it is closed at the beginning of the simulation and remained closed, and in Src, the P-loop is open at the beginning and remains open. Hence, the difference in the dynamics of the P-loop (Figure 7c) is likely due to the different conformations of the P-loop in Src and Abl complexes. The closed P-loop conformation could be less dynamic than the open due to its interaction with the hinge region, an event that also stabilizes the hinge region in the Abl complex (Figure 7e).

In the Src and Abl complexes with imatinib, the αC-helix maintains the active αC-helix-in conformation throughout the MD simulations (Figure 7f), indicating a lack of significant conformational transition in this region. The DFG motif in Abl and Src imatinib complexes is in the inactive DFG-out conformation and remains in this conformation during the MD simulations, as indicated by the low RMSD values of DFG (Figure 7g). No conformational changes were observed in the catalytic loop containing the HRD motif (Figure 7h).

Since other regions in Src and Abl proteins may show altered protein dynamics, we performed RMSD analysis per amino residue. However, this analysis has not revealed other regions that undergo large conformational transitions (Figure S2). Therefore, the difference in imatinib affinity toward Src and Abl may be attributed to the P-loop conformation. In Abl, the P-loop closed conformation traps the imatinib inside the structural confines of the enzyme, and in Src, the open P-loop conformation could be more permissive for its dissociation. 

In most structures of the Abl kinase domain, deposited in the protein databank, the P-loop is in the open conformation. This has led to the hypothesis that imatinib induces the closed P-loop conformation [21,22,23]. However, more recent NMR structures have indicated that Abl has an intrinsic capacity to adopt the open and the closed P-loop conformation, as both conformations were identified in Abl Apo-enzyme in the absence of any ligands (Figure S3) [29].

**Figure 8:**
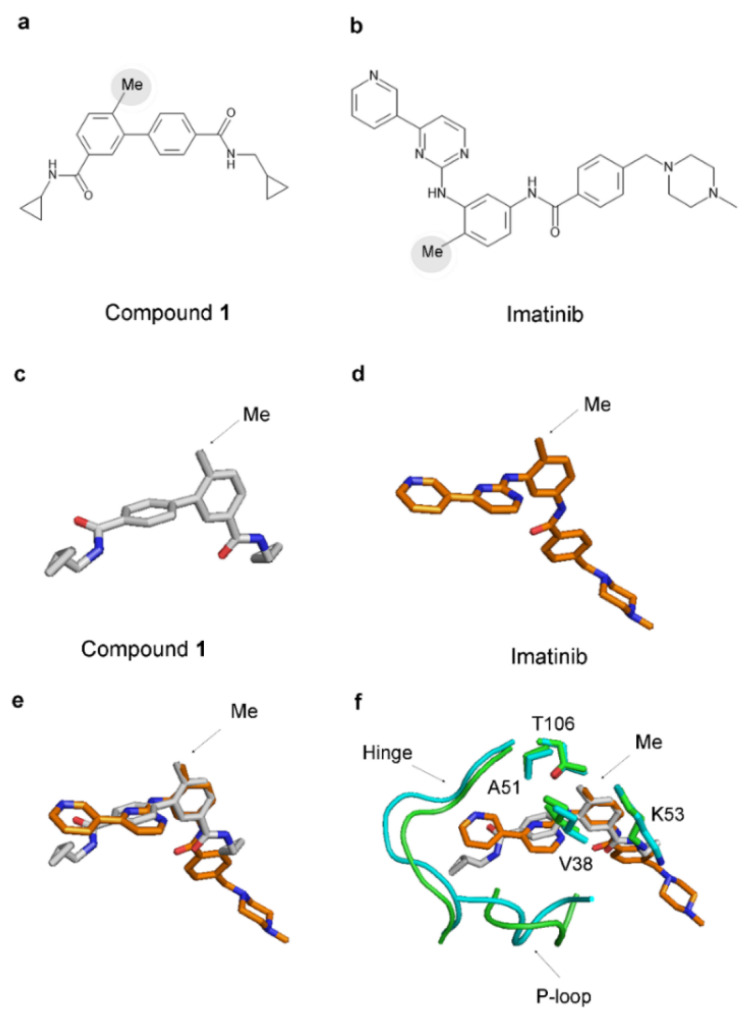
A ‘magic’ methyl is present in the structure of imatinib. (**a**,**b**) The 2D structure of compound **1** and imatinib. Methyl groups are indicated by grey circles. (**c**,**d**) The structure of compound **1** and imatinib observed in their complexes with p38α (PDB 3D7Z) and Abl (PDB 1IEP). (**e**) The superimposition of the structures of compound **1** (grey) and imatinib (orange). (**f**) The superimposition of the binding pockets of compound **1** and imatinib. p38α is in green, and Abl is in cyan. The residues in the hydrophobic pocket binding the methyl group are conserved in p38α and Abl. Numbering is in p38α. V38, A51, K53, and T106 correspond to V255, A269, K271, and T315 in Abl.

The 2D structures of imatinib and compound **1** exhibit notable differences (Figure 8). However, remarkably, imatinib possesses an analogous methyl group akin to the ‘magic’ methyl found in the p38α complexes (Figure 8). This methyl group is attached to the central benzene ring of imatinib (as illustrated in Figure 8b) and, when forming a complex with Abl (PDB 1IEP), occupies an equivalent hydrophobic pocket comprising V255, A269, K271, and T315 in the Abl protein (corresponding to V38, A51, K53, and T106 in p38α) (Figure 8f).

**Figure 9:**
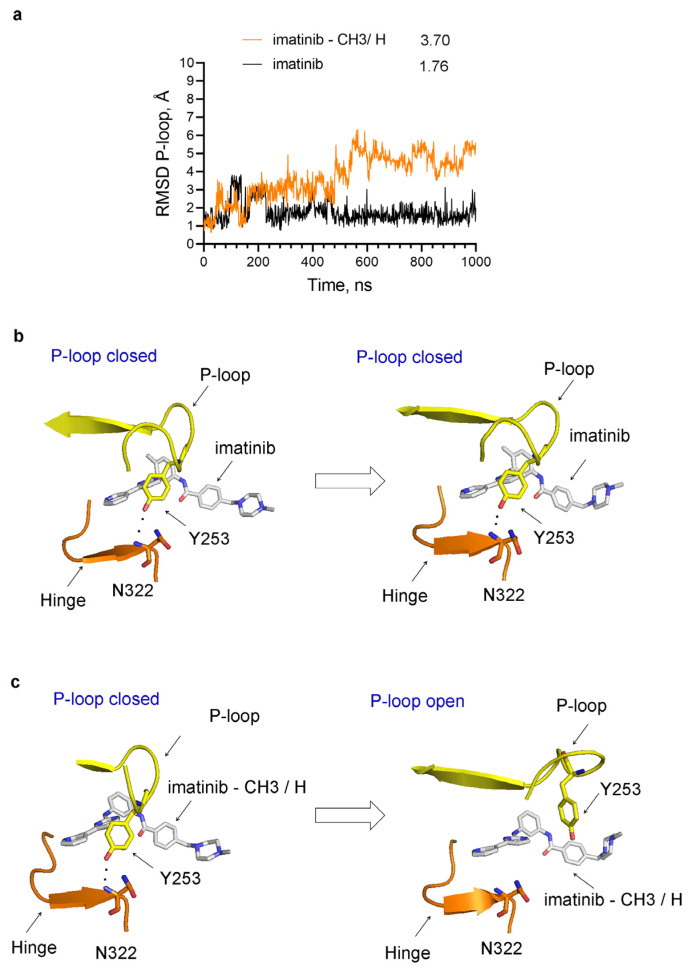
Conformational changes in the P-loop in the complexes of Abl with imatinib alone or imatinib in which the methyl group is substituted with a hydrogen atom (imatinib–CH3/H) during MD simulations. (**a**) RMSD of the heavy atoms of the P-loop in Abl complexes. The numbers next to the legend indicate the average RMSD in Angstroms (Å). (**b**) In imatinib–Abl complexes, the P-loop is in the closed conformation at the beginning and remains closed during the MD simulations. (**c**) The P-loop opens during MD simulations of Abl complexes with imatinib–CH3/H. The hydrogen bond between Tyr253 in the P-loop and Asn322 in the hinge is indicated by black dots. In “(**a**)” the P-loop opens at around 500 ns and remains open until the end of the simulation.

This intriguing revelation suggests that the benzene ring with the attached methyl group serves as a novel pharmacophore, potentially contributing to the stabilization of the closed P-loop conformation and participating in the entrapment of the inhibitor within the protein structure. To test this hypothesis, we performed MD simulations of Abl complexes either with imatinib alone or with imatinib in which this methyl group is substituted with a hydrogen atom (Figure 9). The results have revealed that the absence of the methyl destabilizes the closed P-loop conformation (Figure 9), resembling the previous results for p38α inhibitor complexes (Figure 3 and Figure 4).

Similar results were obtained when the imatinib methyl group was substituted with a fluorine atom or trifluoromethyl group (Figure S4), indicating that the methyl group is required to stabilize the closed P-loop conformation. The methyl group of imatinib does not interact directly with the P-loop, raising questions about the mechanism by which it exerts its effect. One possible explanation is that the methyl group exerts conformational restriction in the imatinib molecule, which, in turn, blocks the conformational change of the P-loop. For example, in imatinib, the hydrogen atoms of the methyl group repulse with the hydrogen atom bonded to the nearby nitrogen donor atom, leading to near-perpendicular orientations of its benzene and pyrimidine rings (Figure S5a,b). The substitution of the methyl group with a hydrogen atom allows for a decrease in the angle between the two rings (Figure S5c). When the methyl group is substituted with a fluorine or a trifluoromethyl group, intramolecular hydrogen bonds that facilitate this conformational conversion of imatinib are formed (Figure S5d,e). 

**Figure 10:**
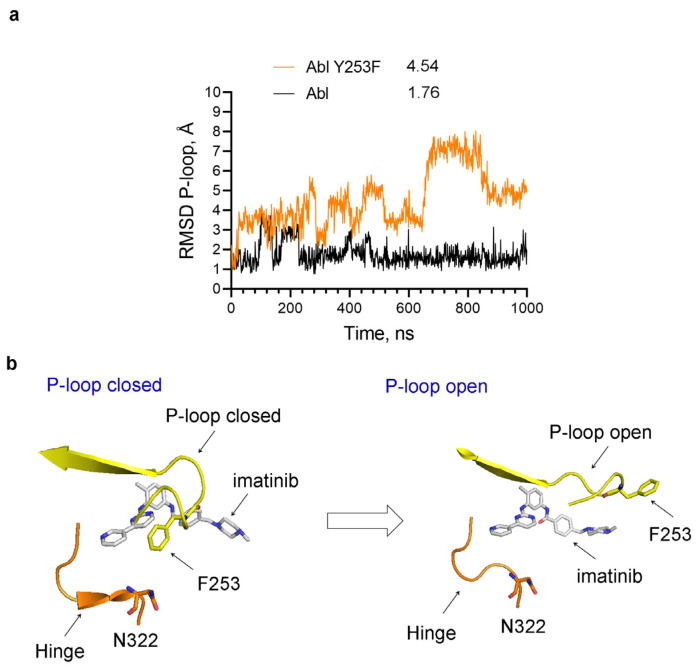
Increased P-loop conformational dynamics in the imatinib–Y253F Abl complex. (**a**) RMSD of the heavy atoms of the P-loop in imatinib complexes with wt Abl (Abl) and Abl containing Y253F mutation. The numbers next to the legend indicate the average RMSD in Angstroms (Å). (**b**) In the Y253F mutant, initially, the P-loop is in the closed conformation and opens during the MD simulations.

In CML patients treated with imatinib, resistant mutations in the Abl kinase domain arise. Among them are mutations in the P-loop, including the critical Tyr253 residue [30,31,32]. For example, the mutation of Tyr253 to phenylalanine (Y253F) leads to very high resistance to imatinib inhibition [30,31,32]. Considering this residue’s central role in forming and stabilizing the closed P-loop conformation, we performed MD simulations to observe how this mutation affects the dynamics of the P-loop in the Abl imatinib complexes (Figure 10). Indeed, we find that the Y253F mutation destabilizes the closed P-loop conformation and allows its transition to open conformation (Figure 10), consistent with the substantial decrease in the binding affinity of imatinib toward this Abl mutant protein.

## 3. Discussion

The imatinib’s high affinity and selectivity for Abl over Src and the methyl effect observed in inhibitors of p38α could be explained by the trapping of the inhibitors inside the protein structure. Both cases are mediated by a similar mechanism involving the closed conformation of the P-loop. In this conformation, the P-loop encloses more tightly around the inhibitor, sealing it from the environment and effectually capturing it inside the protein structure. Tyr253 in the P-loop (Abl numbering) forms a hydrogen bond with Asn322 at the hinge region, which encloses the small molecule inside the protein. The dissociation of the trapped ligands requires a conformational change in the P-loop, and this is expected to come with an energetic penalty required for the disruption of the interactions with Tyr253, as well as for the conformational transition to the open P-loop conformation, which may necessitate rearrangement in other parts of the kinases domain. The difference in the affinity of imatinib against Src and Abl and the ‘magic’ methyl effect in p38α suggest that the energetic barrier for the conformational change necessary for the dissociation of the inhibitor may contribute hundreds or even thousands of fold to the overall affinity (Figure 2 and Figure 5). The described mechanism resembles the inhibitor trapping in the N-myristoyltransferases, where the Ab-loop performs an analogous function to the P-loop in kinases [1]. The NMT inhibitors bind when the Ab-loop is open. Subsequent to its closing, interactions with inhibitors prevent the reopening of the Ab-loop, capturing the inhibitor inside the protein structure [1]. Similarly, imatinib and compound **1** may bind to an open kinase conformation, followed by a conformational change to the closed form. The entrapment of the inhibitor may occur due to blocking the transition to the open P-loop conformation, locking the ligand inside the protein structure. In agreement, in the p38α inhibitor complex, removing the methyl group from the ligand leads to a conformational transition to the open P-loop conformation and a more than 208-fold decrease in affinity. In the crystal structure of the Abl–imatinib complex, the P-loop is in the closed conformation, and in the Src–imatinib complex, the P-loop adopts the open conformation. Hence, in Abl, the imatinib is trapped, and in Src, it is not, explaining the substantial difference in affinity. 

Is there experimental evidence supporting the inhibitor trap model? Kinetic studies have shown that imatinib affinity to Abl is 3000-fold higher than Src [23]. Imatinib binds ten times faster and dissociates 100-fold slower in Abl than Src [23]. Together, the binding and dissociation steps contribute to a 1000-fold difference in affinity, and the remaining 3-fold difference could be attributed to other conformation transitions, such as DFG flipping before the binding of the drug [23]. Notably, the most significant contributions to the difference in affinity come from the slow dissociation step, consistent with inhibitor entrapment.

In the span of over two decades, as new crystallographic structures and experimental data have emerged, different hypotheses have been proposed to explain the selectivity of imatinib for Abl over Src, including a DFG flip and conformational selection between active and inactive protein conformations [23,24]. The closed P-loop conformation in Abl and the open P-loop conformation in Src have been previously observed (referred to in the literature as kinked and stretched conformation) [23,33]. However, initially, the different P-loop conformations were not believed to impact imatinib affinity significantly because they have a minimal effect on the direct interactions between the drug and the Abl and Src proteins. The induced-fit model is the most recent hypothesis, according to which imatinib binding triggers a conformational change in the Abl-kinase domain but not in c-Src [23,33]. In agreement with this model, NMR studies have indicated that a conformational change accompanies imatinib binding to Abl [23]. In addition, kinetics studies have shown that imatinib binding is biphasic—the initial fast binding is followed by slow binding attributed to this conformational change [23]. Most recently, the conformational change during the binding of imatinib was modeled via MD simulations [33]. Although there is substantial evidence for the conformational change in Abl after imatinib binding, it is not certain whether imatinib induces this conformational change or stabilizes a conformation that Abl inherently tends to adopt [33]. In this light, recent NMR structures have revealed that the P-loop of Abl can assume both the closed and open conformations in solution, even in the absence of any inhibitors [29]. Together, these experiments provide evidence supporting the validity of the inhibitor trapping model.

The entrapment of imatinib in Abl is also evidenced by the appearance of resistant mutations in patients treated with imatinib that affect the P-loop and the critical Tyr253 [30,31,32]. Interestingly, the mutation of Tyr253 to phenylalanine leads to very high resistance to imatinib, and biochemical assays have shown that the mutation substantially reduces the imatinib affinity [32]. The results reported here indicate that the Y253F mutation destabilizes the closed P-loop conformation and facilitates its opening. This implies that the hydrogen bond between the hydroxyl group of Tyr253 and the hinge region is required for the high binding affinity of imatinib. Since this interaction occurs between the hinge and the P-loop in the kinase domain and not between the inhibitor and the protein, these findings provide direct evidence for the role of the closed P-loop conformation in imatinib entrapment.

An intriguing consequence of drug entrapment is the high contribution to the binding affinity of the bonds or interactions that are involved in the trapping mechanism. For example, in the p38α complex, the methyl group of compound **1** increases affinity by over 200-fold; in the Abl Y253F mutant, the loss of a single hydrogen bond leads to a dramatic loss of affinity and resistance to imatinib. Therefore, minimal changes in the inhibitor or the protein structure could have a profound effect on binding affinity. The novelty of this study is that it establishes a connection between the binding affinity and the conformational transitions in the protein structure, which may serve as a basis for the development of innovative approaches in drug design. 

The observation that the addition of a single methyl group to the structure of an inhibitor could result in dramatic increases in binding activity has remained enigmatic [27,28]. The results of this study suggest that the methyl group’s role is to restrict the conformational transitions in the protein structure. Specifically, in p38α and Abl, it blocks the transition from closed to open P-loop conformation, which is required for the dissociation of the inhibitor. While the effect of the methyl group on the binding affinity of p38α inhibitors is clearly described in the literature [27], the role of the equivalent methyl group in imatinib is less clear. For example, it has been reported that the development of imatinib started, in fact, as an attempt to design inhibitors of protein kinase C (PKC). Along this process, the methyl group was introduced in a precursor intended as a PKC inhibitor [13]. However, this unexpectedly led to altered selectivity—it eliminated the activity toward PKC but sustained the capacity to inhibit Abl kinases [13].

There is evidence that a chlorine atom may, at least in part, substitute for the methyl group in certain kinase inhibitors. For example, substituting the methyl group of compound **1** with a chlorine atom reduces the affinity by ~2-fold, compared to over 208-fold when substituted with a hydrogen atom or 38-fold when substituted with a fluorine atom [34]. This outcome may be due to the fact that the methyl group and the chlorine atom are of similar size. The idea that a chlorine atom can substitute for the methyl group is also suggested by the Abl inhibitor PD173955. In the crystal structure of PD173955 in the complex with Abl (PDB 1M52) [35], the P-loop is closed, and one of the two chlorine atoms of the compound inserts into the same pocket occupied by the methyl group in the imatinib–Abl complex. 

The ‘magic’ methyl effect in p38α has been proposed to occur due to a conformational restriction in the structure of the small molecule [27]. The methyl group in compound **1** is positioned in one of the two central benzene rings of the molecule (Figure 8a). In this position, the methyl group, due to steric hindrance, is expected to prevent the planar position of the two benzene rings, which are oriented nearly perpendicularly in the crystal structures (Figure 8). This kink in the inhibitor structure may be more supportive of the closed conformation of the P-loop. Notably, the absence of the methyl group does not prohibit the inhibitor from adopting this unique conformation. However, without the methyl group, increased rotational freedom is anticipated between the two benzene entities of the compound. Such molecular dynamism may augment protein dynamics and potentially facilitate a conformational shift to the open P-loop conformation. In the literature, instances are documented where the ‘magic’ methyl effect is observed in inherently rigid molecules [27]. Such examples underscore the likelihood that the effect arises from the influence of the methyl group on the protein conformation and not solely from the conformational restriction of the ligand.

Why does the P-loop in the Src–imatinib complex not adopt the closed conformation seen in the Abl complex? One explanation is that Tyr253 in Abl is substituted with phenylalanine in Src, thus preventing hydrogen bond formation with the hinge region. Evidence that this is a contributing factor comes from the observation that the Y253F mutation in CML patients leads to imatinib resistance [32]. However, this alone does not explain the differences between Src and Abl because substituting the phenylalanine residue for tyrosine in Src does not lead to a high affinity to imatinib [22]. Studies of ancestral to Src and Abl kinases have attributed the differences to 15 amino acid residues [24]. These amino acid differences include Tyr253 and several others located in the P-loop and the hinge region [24]. However, most differences occur in the N-lobe of the kinase domain, far away from the imatinib binding site [24]. The fact that residues distant from the binding site of imatinib affect its affinity can be explained by considering their role in modulating the protein dynamics within the N-lobe, affecting the probability of conformation transitions in the P-loop.

When examining different crystal structures, it is evident that the protein tends to crystallize in a singular conformation that depends on the structure of the inhibitor. Notable instances include how Abl crystallizes in the inactive DFG-out conformation with imatinib and in the active DFG-in conformation in the complex with dasatinib, and the epidermal growth factor receptor (EGFR) crystalizes with erlotinib in the active αC-helix-in conformation and in the inactive αC-helix-out conformation in the presence of lapatinib [21,36,37,38]. Given that the kinase domain can transit from one conformation to another in solution, these findings indicate that the inhibitors select and trap a specific protein conformation. Intriguingly, this conformational selection could be, in fact, a consequence of the inhibitor’s entrapment within the protein structure. This is because entrapping the small molecule necessitates a limitation in the protein’s conformational transitions, thereby capturing the protein in a distinct conformational state. Thus, the inhibitor-trapping mechanism is inherently bidirectional in its effects. While the inhibitor is entrapped in the protein structure, reciprocally, the protein is simultaneously ‘captured’ within a specific conformational state. 

In the cases of imatinib and compound **1**, the ability to stabilize the P-loop’s closed conformation proves crucial for inhibitor potency. Yet, this does not discount the significance of other structural elements in influencing drug affinity. In our studies of NMTs, it has been apparent that inhibiting the conformational mobility of multiple structural components simultaneously is necessary for efficient entrapment [1]. Remarkably, the crystal structures of the complexes between imatinib and Abl, or compound **1** and p38α, demonstrate that these inhibitors engage all major conformational mobile elements of the kinase domain, including the DFG motif, the αC-helix, the hinge, and the P-loop (Figure S6), potentially blocking their movements. This underscores the idea that achieving high-affinity binding requires a ligand that impedes the mobility of all conformational components, and a failure in this regard, as demonstrated for the P-loop in this study, might substantially compromise affinity.

The inhibitor trapping has a profound effect on the affinity of the inhibitors, as observed in Abl, p38α, and NMTs [1,24], and, hence, it is expected to play a critical role in the therapeutic activity of these compounds. Interestingly, the trapped inhibitors exhibit remarkable affinity, reaching into nanomolar or even picomolar levels, whereas compounds not subject to trapping typically display micromolar activity [1]. This raises the intriguing question of whether inhibitor trapping is invariably required for high-affinity binding and potency. For example, in the Src and Abl imatinib complexes, the interactions between the protein and the drug are conserved, suggesting that the direct interactions between the inhibitor and the protein contribute to micromolar activity (Ki = 30 µM for the Src–imatinib complex) and that nanomolar affinity is achieved through the trapping mechanism (Ki = 10 nM in the Abl–imatinib complex). Considering the diverse array of proteins and the plethora of potential ligands, a definitive answer to this question demands a considerable research effort.

The discovery of inhibitor trapping in the vast family of protein kinases, in addition to NMTs, suggests that this might be a frequent or even ubiquitous mechanism. Lending further credence to the idea that the ‘magic’ methyl effect has been noted in a variety of proteins besides the kinase family, including G-protein-coupled receptors and enzymes [27,28]. In addition, a similar disproportionate high contribution to affinity has been seen for other bonds, including salt bridges, hydrogen bonds, halogen bonds, and stacking interactions, and their effects could be analogous to that of the methyl group [3,26]. In addition, inhibitor trapping could be the mechanism responsible for tight binding inhibition [1]. This inhibition is characterized by high affinity due to the ligand’s very low dissociation rate. The NMT inhibitors IMP-1088 and DDD85646, as well as imatinib, belong to this group [1,23]. Tight binding inhibitors are also observed in many other protein targets, including JAK kinases, COX-1/2, *E. coli* DHFR, *M. tuberculosis* enoyl reductase, and others [33,39,40], also implying that inhibitor trapping occurs in diverse proteins.

Inhibitor trapping can also determine drug selectivity because the same ligand can become trapped in some target proteins but not in others, resulting in dramatic differences in affinity, even when the binding sites are highly conserved. Examples include the selectivity of imatinib for Abl over Src and the preference of imatinib to Abl kinase over protein kinase C due to the introduction of the methyl group. In NMTs, the trapping allows for the selective targeting of the protozoan NMTs over the human counterparts, although they have near identical binding sites [25]. The model of inhibitor trapping suggests that selective targeting of conserved binding sites is feasible due to amino acid differences outside the binding site that influence the protein dynamics and the ability of a specific ligand to become entrapped in one structure but not in others. This approach might also prove valuable for selectively targeting kinase family members, especially given the high conservation of the ATP binding pocket across its 500 members.

The inhibitor trap model introduces a new perspective in drug design, offering a different approach to developing high-affinity pharmaceuticals. It underscores the notion that during drug development, there should be a strategic focus on designing compound structures with the intention of blocking the protein’s intrinsic conformational movements. This process can be helped by developing artificial intelligence (AI) approaches. Given the findings from the present study, the AI-based methodologies aimed at forecasting inhibitor affinity might necessitate an emphasis on predicting protein conformations akin to Alphafold [41] or may be based on databases of molecular dynamics simulations of protein–ligand complexes. The current limitation of this approach lies in the challenge of utilizing computational methods to propose compound structures effectively inhibiting protein conformational changes and resulting in entrapment. Presently, aside from MD simulations, no other computational methodologies address inhibitor trapping, and most computational scientists may not even be aware of its existence. Predicting it may necessitate the development of innovative tools, programs, and algorithms, ultimately reshaping the landscape of highly potent inhibitor design in the future. This development could also be facilitated by the recent substantial interest in linking AI models with molecular dynamics to improve the speed, forced field performance, and analysis of simulations [42].

## 4. Materials and Methods

Visualization of protein–ligand interactions and image preparation. Protein–ligand interactions were visualized using PyMOL 1.6.0.0 (Schrödinger, New York, NY, USA) [43] and YASARA v. 20.4.24 (IMBM, University of Graz, Graz, Austria) [44]. Surface representations of the complexes were visualized using PyMOL 1.6.0.0 (Schrödinger, New York, NY, USA). 

Preparation of proteins and ligands for MD simulations. MD simulations were performed using the crystal structures of the Abl–imatinib complex (PDB 1IEP), Src–imatinib complex (PDB 2OIQ), and p38α-–compound **1** complex (PDB 3D7Z). The p38α–compound **2** complex was obtained after removing the methyl group from compound **1** using PyMOL. Similarly, the substitutions of the imatinib methyl group were performed using PyMOL. The Y253F mutation in Abl protein was introduced using PyMOL (Schrödinger, New York, NY, USA). The crystal structures of the Abl–imatinib complex (PDB 1IEP) and p38α–compound **1** complex (PDB 3D7Z) do not contain disordered regions. In the crystal structure of the Src–imatinib complex (PDB 2OIQ), part of the A-loop was disordered and repaired via homology modeling using Maestro (Schrödinger, LLC, New York, NY, USA). The protein in the p38α–compound **1** complex (PDB 3D7Z) contains S-hydroxycysteine at position 119. The presence of S-hydroxycysteine in the proteins is an artifact due to X-ray irradiation during crystal structure determination. That is why the S-hydroxycysteine was converted to a cysteine residue using PyMOL. N-terminal ACE CAPs were added to Src and Abl proteins to block the positively charged N-termini generated because of the truncation of the crystalized proteins and not present in the full-length protein. C-terminal NME CAP was added to the Abl and p38α proteins to cap the unnatural negatively charged C-terminus created by the truncation of the proteins used to generate the crystal structures. The inhibitor structures were parametrized using the GAFF2.11 force field and AM1-BCC charges using Antechamber from the Amber v.18 package [45,46]. 

Molecular dynamics simulations. MD simulations were performed as previously described [47,48]. In brief, the complex was solvated in saline (0.9% sodium chloride) in a truncated octahedral box; energy minimized; heated to 310 K at constant volume for 1 ns; density equilibrated at 1 bar for 1 ns; equilibrated keeping constant T and *p* for 1 ns, using the Langevin thermostat [49] and Berendsen barostat [50]; and simulated for 1000 ns using AMBER v. 18 (UCSF, San Francisco, CA, USA) [51,52]. During all steps of simulations, i.e., heating, density equilibration, preproduction, and production, the dynamic SHAKE algorithm was used for constraining covalent bonds involving hydrogen with a 2 fs time step [53]. The bonds to hydrogen were not constrained only during the energy minimization step. The systems were simulated with the ff14SB force field [54] under periodic boundary conditions. Frames were saved every 1 ns to generate 1000 frames for a 1 µs duration of MD production simulations. The duration of the MD simulations was appropriate for the observation of conformational movements in the protein structure. 

Analysis of MD simulations. The data from the MD simulations were analyzed using VMD (Visual Molecular Dynamics, the University of Illinois at Urbana-Champaign, IL, USA) [55]. RMSD (root-mean-square deviations) values were determined using the RMSD trajectory tool in VMD. RMSDs of the protein, ligand, αC-helix, DFG, hinge, P-loop, HRD–loop, and A-loop were determined after performing alignment (superimposition) for the protein in all the frames of the simulations in VMD. Thus, the RMSD values reflect the relative positions of the studied elements relative to the protein, i.e., their conformational movement. RMSF (root-mean-square fluctuation) was not determined, as it was not optimal for examining the mechanism of inhibitor trapping. While RMSD quantifies deviations from aligned superimposed structures, RMSF measures the fluctuation of atom positions around their average locations over time. RMSF often aligns more closely with protein secondary structures: loop regions typically exhibit higher RMSF than α-helices and β-sheets. For instance, if a helical region undergoes a conformational shift yet maintains its secondary structure, its RMSD will increase significantly, whereas the RMSF of its atoms will remain low.

Given that inhibitor trapping is contingent on protein conformational shifts, RMSD provides more insightful data for such analysis. RMSD per residue was determined after aligning the protein in all frames of the MD simulations and determining the RMSD of the Cα-atoms for each residue of the proteins using VMD. For visualizing the differences in RMSD of each residue in the protein structures, the RMSD values of each corresponding residue of the two compared proteins (for example, Src and Abl) were subtracted, and the obtained values were introduced in place of the B-factors of the Cα-atoms in the pdb file. Coloring, based on the B-factors of the Cα-atoms, using PyMOL produced figures in which the regions with slight differences in RMSD are depicted in blue, with the middle differences in yellow and the high differences in red. 

## Data Availability

Data contained within the article.

## Supplementary Materials

The following supporting information can be downloaded via this link: https://www.mdpi.com/article/10.3390/ijms25063249/s1.
