# Supplementary material for: Inhibitor Trapping in Kinases
Source: Int J Mol Sci. 2024 Mar 13;25(6):3249. doi: 10.3390/ijms25063249 (PMC10970472; doi:10.3390/ijms25063249)
Supplement: Supplementary file 1 [file ijms-25-03249-s001.zip › ijms-2872227-supplementary.pdf]

Supplementary Materials

Inhibitor trapping in kinases

Danislav S. Spassov *, Mariyana Atanasova and Irini Doytchinova

Department of Chemistry, Faculty of Pharmacy, Medical University of Sofia, 1000 Sofia, Bulgaria; matanasova@pharmfac.mu-sofia.bg (M.A.); idoytchinova@pharmfac.mu-sofia.bg (I.D.)
* Correspondence: dspassov@pharmfac.mu-sofia.bg

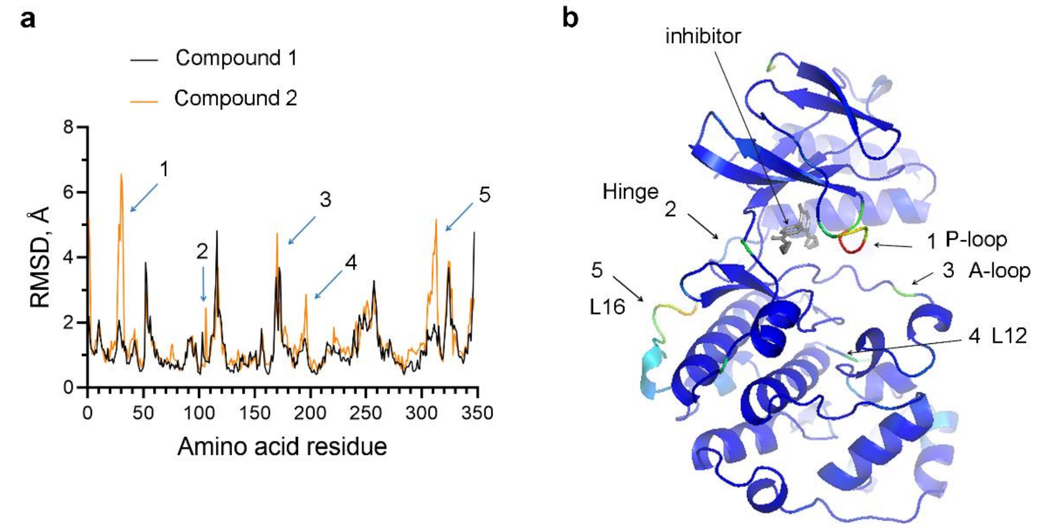

Figure S1. Conformational dynamics of p38α in complex with compound **1** or **2** (**a**) Average RMSD for the alpha carbon atoms for each amino acid residue during MD simulations of the p38α protein in complex with compound **1** or compound **2**. Arrows and numbers indicate regions with increased dynamics in compound **2** vs. compound **1** complex. The most dramatic difference is seen in the P-loop (peak 1); (**b**) The crystal structure of p38α based on PDB 3D7Z, depicting the differences in dynamics in compound **1** and compound **2** complexes. The differences in dynamics increase from blue to yellow and red. Numbers correspond to the numbers in figure a. Large conformational transitions occur in the P-loop (peak 1) and L16 (peak 5).

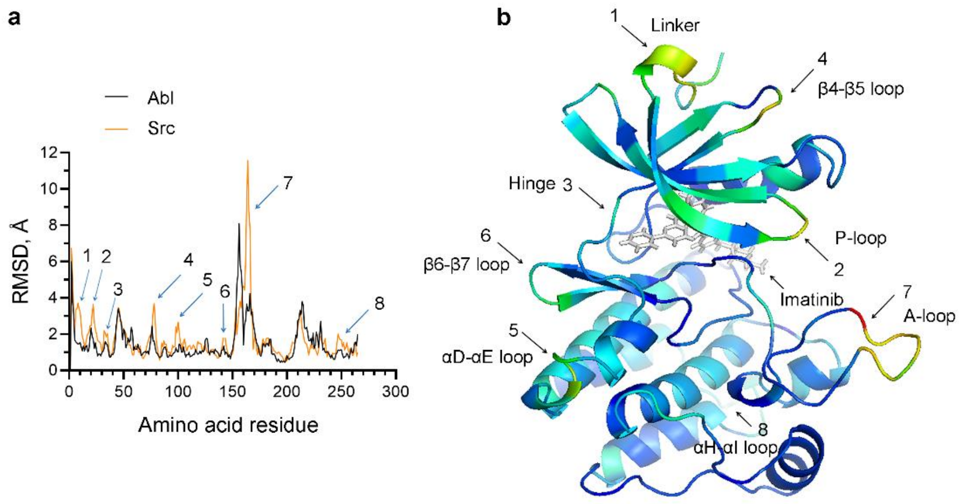

Figure S2. Conformational dynamics of Abl and Src complexes with imatinib. (**a**) Average RMSD for the alpha carbon atoms for each amino acid residue during MD simulations of the Abl and Src protein in complex with imatinib. Arrows and numbers indicate regions with increased dynamics in Src compared to Abl complexes; (**b)** The crystal structure of Src, based on PDB 2OIQ, depicting the differences in dynamics between Src and Abl complexes. The differences in dynamics increase from blue to yellow and red. Numbers correspond to the numbers in figure a. The Src protein has increased dynamics compared to Abl; however, more substantial conformational transitions are absent.

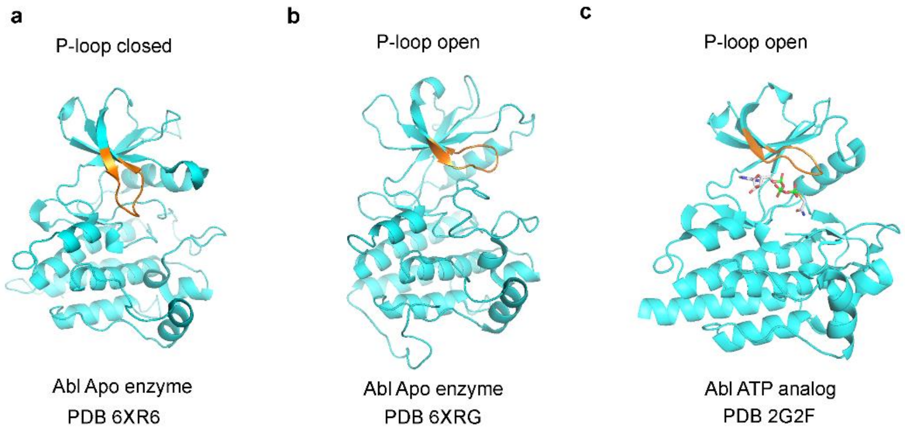

Figure S3. Abl has an intrinsic capacity to adopt the closed and the open P-loop conformation. (a) NMR structure of Abl in the absence of ligands with the P-loop in closed conformation; (b) NMR structure of Abl in the absence of ligands with the P-loop in open conformation; (c) Crystal structure of Abl in complex with non-hydrolyzable ATP analog. The P-loop is in open conformation and interacts with the phosphate groups of the ligand.

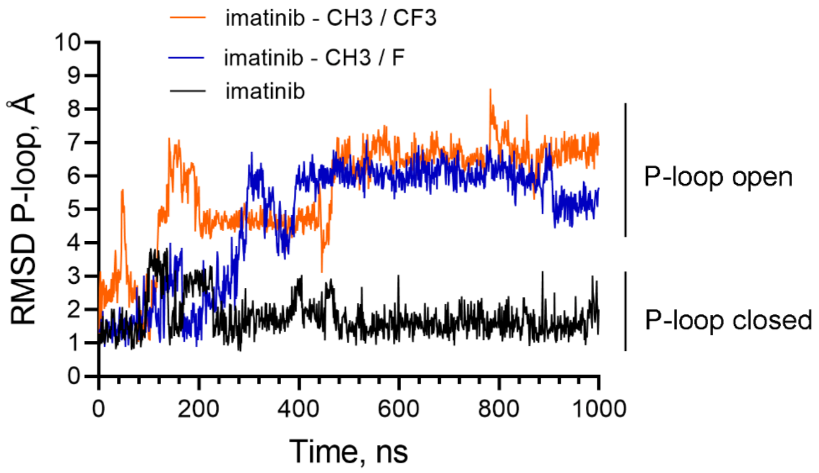

Figure S4. RMSD of the P-loop heavy atoms in the complexes of Abl with imatinib or imatinib in which the methyl group is substituted with a fluorine atom (imatinib – CH3 / F) or trifluoromethyl group (imatinib – CH3 / CF3). The P-loop opens at around 120 ns and 280 ns in imatinib – CH3 / CF3 and imatinib – CH3 / F Abl complexes, respectively, and remains open until the end of the simulations.

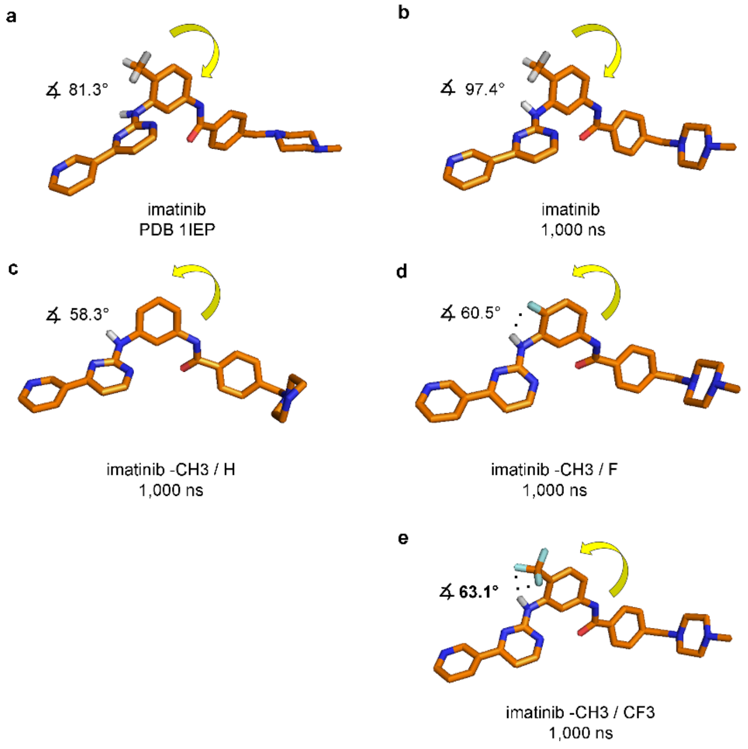

Figure S5. Conformational restriction in the imatinib structure. (**a**) The structure of imatinib in its complex with Abl (PDB 1IEP); (**b**) The structure of imatinib after 1,000 ns of MD simulation of its complex with Abl; (**c**) the structure of imatinib, in which the methyl group is substituted with a hydrogen atom after 1,000 ns of MD simulation of its Abl complex; (**d**) the structure of imatinib, in which the methyl group is substituted with a fluorine atom after 1,000 ns of MD simulation in its complex with Abl; (**e**) the structure of imatinib, in which the methyl group is substituted with a trifluoromethyl group after 1,000 ns of MD simulation in its complex with Abl. The dihedral angle between the benzene ring containing the methyl group and the pyrimidine ring is shown. The substitution of the methyl group allows a decrease in the angle between the two rings. Black dots indicate intramolecular hydrogen bonds.

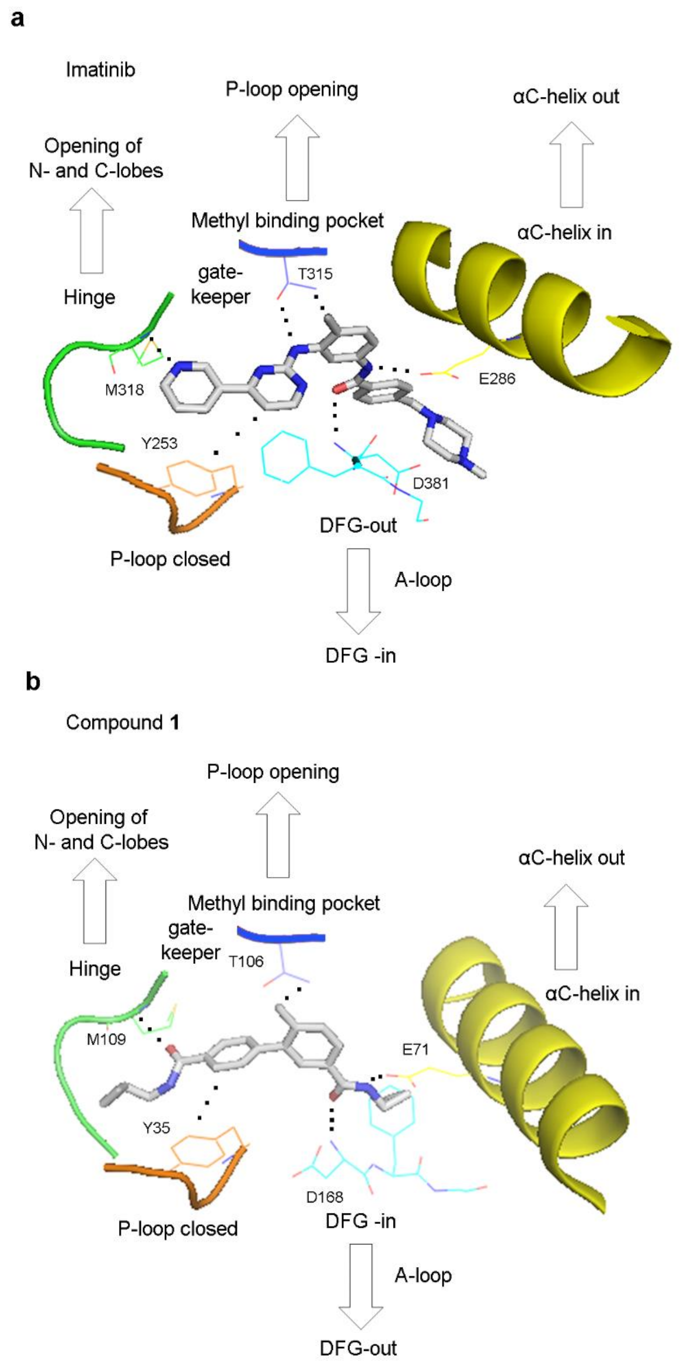

Figure S6. Inhibitor potency may depend on restricting the conformational movement in the protein structure. (**a**) The binding site of imatinib in Abl (PDB 1IEP). The conformation is DFG-out, αC-helix--in; (**b**) The binding site of compound **1** in p38α (PDB 3D7Z). The conformation is DFG-in, αC-helix-in. The interaction involved conserved amino acid residues present in Abl and p38α proteins, including glutamic acid from the αC –helix (E286 in Abl, E71 in p38α), the backbone amide group of aspartic acid residue from the DFG motif (D381 and D168, respectively), the backbone amide group of methionine residue in the hinge region (M318 and M109). Both compounds' 'magic' methyl inserts into a hydrophobic pocket partly formed by the conserved gatekeeper residue (Thr315 and Thr106 in Abl and p38α). The conformational changes known to occur in kinases, such as the movement of the αC-helix, DFG flip, the opening and closing of the N-and C-lobes of the kinase domain through the hinge region, and the transition from the closed to open P-loop conformation are indicated by arrows. Note that the depicted interactions (black dots) are expected to restrict all these conformational movements.
